# Clinique Des Maladies Des Enfans Nouveau-nés

**Published:** 1839-03-09

**Authors:** 


### BIBLIOGRAPHICAL NOTICE.

*Clinique des Maladies des Enfans nouveau-nés.* Par F. L. J. Valleix, D. M. P. &c. Paris. 1838. 1 vol. 8vo. pp. 684.

*A Clinic of the Diseases of Infants.* By F. L. J. Valleix, D. M. P., &c.

The author of this volume is one of the most distinguished disciples of the School of Louis, and resembles that great teacher in his love of truth and his aptitude for the methodical observation of facts. As the work before us lays no claim either to elegance of diction, profound learning, or that vividness of imagination, which is the parent of bold hypothesis, but is, on the contrary, a plain and practical statement of facts observed with skill, and recorded with clearness and honesty, we feel that Dr. Valleix has a right to something more than those general and unmeaning terms of praise or blame which may be predicated of almost any work, and which save the reviewer so much tedious labour. We shall make, then, such an analysis of the "*Clinique*" as will present the general reader with its most interesting features, do justice to its author, and consist with the limits assigned for our notice.

Dr. Valleix, after paying a just tribute to the treatise of Billard upon the diseases of infants, regrets that that author should have applied a strict method of analysis only to the portion of his subject which treated of pathological anatomy, and claims that his own labours have been productive of more valuable fruits, he having studied "not merely fragments of diseases, but the diseases themselves, i. e. the symptoms, their order, the necroscopic lesions, and the relations of these several elements to one another."

In a first chapter is shown the difficulty of applying to infants the means of clinical observation in use for adults; the necessity of studying the modes of expression peculiar to the infantile age, the cries, movements, &c., how far these signs are accurate interpreters of the state of the organs and their functions, and finally what is the healthy condition of these last, without which knowledge it is impossible to know when a function is deranged. In regard to the face, it is stated that the dark reddish hue of new-born infants begins to diminish about the fourth day, when it is mingled with a slight yellow tinge which shows itself rather on all other parts of the face than over the cheek-bone. If the original red colour persist beyond a week without a shade of yellow we may expect the developement of some disease, and, commonly, œdema. When the yellow tinge appears suddenly and dyes the sclerotica, it is ominous of evil.

In a healthy new-born infant the face is *without expression*, but in acute diseases the features become distorted, the brow wrinkled, the eyebrows contract, and the corners of the mouth are drawn outward. Intermittent movements of the head and limbs, as well as of the features, are no doubt owing to momentary but recurring pains. Dr. V. has only observed them in apththæ and enteritis.

The pulse should be felt, if possible, when the infant is in a state of repose; the physician should follow with his hand the movements of the patient's arm, and not oppose them. Of thirteen infants in perfect health, aged from two to twenty-one days, the maximum of pulsations in a minute was 104, the minimum 76, the general mean 87. Dr. V. has found that the best means of stilling the cries, and calming the agitation of infants who resist physical examination, is to place them before a bright light. The manner in which this agent affects them should be noted.

The next chapter is on *Pneumonia*. Dr. V. has not met with a single case of idiopathic pleurisy, and Dr. Billard only records two: this disease complicates pneumonia in about one-eighth of the cases. The history of the symptoms of the last mentioned disease, as given in the work under consideration, offers no points of novel interest. The mortality from pneumonia at the Foundling Hospital is very great; fifteen cases observed by Dr. V., all terminated fatally, and of 114 noted by Dr. Vernois, one only recovered. It is true, most of these cases were complicated either with aphthæ, erysipelas, or œdema, and of a total of 128 cases, 111 were of double pneumonia. Of this latter number, the disease predominated on the right side 59 times, on the left 10 times; the two sides were equally affected 42 times. Of the 128 cases, seventeen had the right lung exclusively inflamed; in no instance was the left only attacked. In the summer of 1836 we observed, at the Children's Asylum, Blockley, seven cases of pneumonia in patients between the ages of 18 months and two years. In six of these which proved fatal, the right lung only was interested. The remaining case got well: the patient was a robust boy three years old: in him the disease occupied the left lung anteriorly. The lesions found after death by Dr. V. are such as confirm the correctness of Dr. Gerhard's observations, and particularly in regard to the smooth shining aspect of the cut surface of the hepatized lung as contrasted with the granulated state observed under similar circumstances in adults.

The third chapter is occupied by a minute history of "*le muguet*," or aphthæ, a disease so common in the Foundling Hospital of Paris, that about one-fourth of the patients brought to the infirmary of that institution are affected with it. We find that there is some confusion of terms amongst writers on the diseases of children in regard to the name of the disease in question. Dr. Valleix assigns ulceration as the character of aphthæ, while the "*muguet*" is known by him as a membraneous exudation without lesion of the subjacent parts. M. Dugés uses the two words indifferently, and describes ulcerative aphthæ as varieties of the general disease characterized by false membrane. Dr. Robertson says that "aphthæ ought to be considered inflammatory exudations rather than ulcers:" and Dr. Dewees having described under the head of aphthæ the "*muguet*" of the work before us, devotes a subsequent chapter to "ulceration of the mouth." In this he criticises Dr. Underwood for calling "*aphthæ gangrenosa*" a form of ulceration incident to dentition, saying, "the appearances here do not bear the slightest analogy to aphthæ." In this analysis the words aphthæ and *muguet* will be used as synonymous.

Twenty-four cases of aphthæ, observed in their minutest details, form the basis of the memoir before us. "Hitherto pathologists have attended too exclusively to the morbid appearances of the mouth and œsophagus, considering the accompanying affection of the intestinal canal as a complication merely; above all, they have dated the commencement of the disease from the first appearance of false membrane in the mouth." This statement is probably true of the French writers, but neither American nor English authorities are chargeable with such exclusive views. In the "Encyclopœdia of Practical Medicine," Dr. Robertson states that "many authors of reputation contend, that the disease *always originates in the stomach*, even before showing itself in the mouth and fauces." Dr. Dewees expresses himself thus: "This affection (aphthæ) is thought to be *altogether of a symptomatic kind*, or very rarely idiopathic. It is almost uniformly preceded by a deranged condition of the alimentary canal, and always, we believe, by some disturbance of the stomach itself. * * * * * * The bowels are often teased by watery acrid stools of a greenish colour." Dr. Valleix has, however, the credit of demonstrating what others have surmised ; no writer before him has pointed out the succession of the symptoms, nor their mutual relations of frequency, &c., all points of capital importance, but never to be settled by those who write from no better data than are furnished by the most fallacious of all our faculties—an unassisted memory.

The following is an abstract of the results obtained by Dr. V. In seventeen out of twenty-three cases, an erythema of the posterior part of the buttocks preceded the formation of false membrane in the mouth, by six days; in the remaining six cases the erythema existed, but made its appearance later. Diarrhœa, with yellow stools, failed only in one instance to precede the affection of the mouth, and in the great majority of cases, by about four days. At the same time the pulse rose suddenly from 80 or 90 to 116 or 140, and became more developed. The face began to acquire a yellowish tinge, which it preserved throughout the disease. Immediately afterwards the papilla of the point of the tongue began to swell and wear a bright red colour, which extended itself to the cheeks, and within two days and a half was followed by disseminated aphthous points, showing themselves in twenty out of twenty-four cases, first upon the tongue. Upon the cheeks the exudation was deposited in irregular patches; upon the roof of the mouth in layers; at first it was always adherent, and attempts to detach it produced slight hæmorrhagy; later in the disease its removal became easy. During its developement the erythema and diarrhœa persisted; the stools being almost always greenish, but in no instance containing fragments of false membrane. There was moderate tympanitis of the abdomen when the disease was at its height, and evidences of pain upon pressure uniformly corresponding to a lesion of the intestine. Vomiting occurred five times, but in no case was the colour of the membrane of the mouth referrible to the nature of the matter rejected. In several instances the vomiting was clearly caused by the accumulation of false membrane in the posterior fauces. Twenty times there were ulcerations either of the malleoli, or heels. The heat of the surface was notably augmented in twelve cases. "Towards the close of the disease all these symptoms diminished in intensity, and gave place to a state of collapse. The erythema lost its vivid tint; scabs incrusted the ulcerations; the diarrhœa became less abundant or ceased altogether; the aphthæ were limited to a few scattered patches, generally upon the tongue; the pulse fell to 80, 70, or even 60; the warmth of surface was replaced by a coldness, at first confined to the extremities, but subsequently extending itself to the trunk; the agitation gave way to an almost complete insensibility, the cries changed into moanings; the emaciation and paleness became extreme, giving to the face an expression of decrepitude; then, too, there appeared in certain cases sub-acute inflammations of the nose, the lower lip, or the neck, marked by œdematous swellings, obscure redness and pain; abscesses, too, sometimes in large numbers, attacked the subcutaneous cellular tissue; and in one case there was gangrene of the leg: finally, death, without apparent suffering closed the scene."

The average duration of the disease in the fatal cases was seventeen and a half days; in those which recovered, sixteen and a half days. The false membrane, examined after death, presented the following characters:—In no instance did it offer any trace of organization, nor the slightest medium of connexion with the subjacent mucous membrane. On removing from the tongue a portion of recently formed exudation, the papillæ underneath it were found in a state of turgescence, but where the pseudo-membrane was of long standing, the surface below was smooth and dry, and the epithelium intact. No ulcerations existed under the aphthæ, and when observed, they occupied the median line of the hard palate; at times, only superficial, and at others, penetrating to the bone. In ten out of twenty-four cases, they were aphthæ in the pharynx, under the form of small patches, particularly numerous about the edges of the epiglottis. The œsophagus was attacked in seventeen out of twenty-two cases; in nine of these, throughout its whole extent; in eight, the morbid secretion was in the form of zones, one or two inches in height. Under whatever form, the disease stopped short of the cardia by three or four lines. One case only was noted of well defined aphthæ in the stomach, and existed there in the shape of long bands, extending on either side of the lesser curvature, from the cardiac to the pyloric orifice; besides which, there were several patches towards the greater curvature. The small intestine only once presented any traces of pseudomembrane, and then upon two of Peyer's glands nearest the cœcum. This intestine presented, however, almost constant alterations, either in the colour, thickness, or consistence of its mucous coat. The large intestine offered, in about one half of the cases, an increase of redness, generally confined to the rectum. The other organs offered no appreciable lesion but in exceptional cases.

Various causes have been assigned for the production of the "muguet;" but Dr. V. rejects the evidence adduced in support of the doctrine that either premature weaning, bad air and diet, contagion, feebleness of constitution, &c., necessarily expose infants to the attacks of this disease. At the same time he admits the difficulty of obtaining accurate data upon these points, and adds, that whatever the efficient causes may be, they are certainly very active in the Foundling Hospital of Paris, for sporadic aphthæ are rarely met with in the private practice of that city. To superficial observers of disease, the history of causes may doubtless seem very easy to be settled; but the medical analyst knows no subject more perplexing, nor more uncertain in its results. The therapeutic details given by our author are not very satisfactory, but the following deductions are, we think, important:—1st. That unless the patients are withdrawn from the influences under which they have contracted the disease, the most active remedies are inefficacious. 2d. That when this indication is fulfilled, the most simple treatment has been followed by success.

Such is an abstract of the history of *aphthæ*, when well defined; but there are cases of the disease having but an imperfect coincidence with the type. Dr. V. details one in which no evidence of the existence of pseudo-membrane was shown during life, although the general symptoms were characteristic. After death, the œsophagus was found to be these at of the exudation. Three cases are also given under the head of "*Enteritis*," which presented all the abdominal and other signs of aphthæ, except only that neither before nor after death was any secretion of false membrane detected. With these facts before him, the author asks if he may not fairly infer the deposit of false membrane in the mouth and alimentary canal to be one of the many effects, and not the essence of the disease? If this view be admissible, (and we think its correctness nearly demonstrated,) the "*muguet*" may be thus defined:—"A disease characterized at the outset by a marked febrile movement, and an enteritis of greater or less intensity, subsequently by an inflammation of the mouth, with secretion of false membrane, and still later by a similar inflammation of the œsophagus."

The chapter of the work before us which contains the most original matter, and carries with it the most conclusive proof of the author's sagacity and the advantages of the inductive method in medical science, is that entitled "*Cephalæmatome, or bloody tumour of the head.*" The different bloody tumours observed on the heads of infants, soon after birth, may be ranged either among—1st, the sero-sanguineous, or infiltration of the sub-cutaneous cellular tissue, observed after protracted labour; 2d, the sub-aponeurotic, caused generally by external violence, and of rare occurrence; 3d, the sub-pericranial. It is this last variety which demands particular consideration. It is met with in about one out of every four or five hundred births at the "*Maternité*" in Paris. Six cases have been observed by Dr. Valleix. The tumour in question is for the most part observed upon one or the other of the parietal bones, most frequently on the right, and in no case extending beyond the limits of the bone. The tumour usually commences within a few hours after birth; it is at first small, but increases rapidly, sometimes for several hours, or even for two days; the skin over it is of a dark colour, and slightly œdematous; it offers a more or less distinct fluctuation; it is surrounded by a hard ridge a line or two higher than the general surface of the bone; by placing the finger over the ridge, and pressing steadily forward to the centre of the tumour, the bone forming the floor of this latter may be felt. Bearing these characters in mind, the observer may easily distinguish it from any other sanguineous tumour of the head, from hernia cerebri, &c., but the same signs are presented by purulent sacs between the skull and its periosteum. To explain the formation of the disease, the author describes the normal condition, at birth, of the parts subject to attack. "The pericranium is but slightly adherent to the skull in new-born infants, except within a few lines of the several sutures. In the order of developement, the internal table of the skull is first formed; numerous vessels ramifying on its external surface, secrete a rudimental diploe,—on this is gradually deposited a layer of compact matter, which constitutes the external table. At birth, the only points completely ossified in the parietal bones are its protuberances, points whence the greatest number of vessels diverge to the periphery of the bones. Except in these points, the work of ossification is going forward in the other portions of the bone during the first fifteen days after birth, or even for a longer period. At this epoch we have then an internal table, or diploe, but *no external table.* Now, if pressure, and, above all, *circular pressure*, be exerted upon any portion of the imperfectly ossified cranium, the blood will rupture its tender vessels, and be extravasated between the bone and the pericranium, detaching this latter in an extent proportioned to the quantity of blood thrown out, or the force employed. "In my researches on the developement of the skull," says Dr. V., "I was struck with the frequency of an ecchymosis in the upper portions of the parietal regions, of greatest extent on the right side, strictly limited by the sutures, and irregularly oval in its shape. This ecchymosis could not be caused by pressure from a resisting plane surface,—the rounded form of the head precludes such an idea: besides, the lesion in question was on or near the summit of the head, while the points which come in contact with the resisting parts of the pelvis, during labour, are either anterior, posterior, or lateral. We must then conclude that the ecchymosis was caused by a circular pressure, the agent of which is situated in the centre of the parts passed over by the head: this agent can only be the neck of the uterus. As the first position of the fœtus is the most common, during its descent through the the pelvis the right parietal bone is placed more in advance than the left, whence the greater frequency of the lesion upon the right side. It is clear that the same causes which give rise to ecchymosis, may, if exerted with superior energy, produce a serous infiltration, or a proper bloody tumour. The conditions necessary to the production of the cephalæmatome, viz. the presentation of a large surface of one of the parietal bones to the mouth of the uterus, are not often met with. Hence the disease is rare.

The ridge which limits the tumour is an osseous formation, elevated about a line and a half (French) above the general surface of the bone, from which it may be detached by insinuating either the nail or a scalpel under it. The bone below offers no change from its natural healthy appearance. The form of the ridge is constantly triangular; its composition varies in regard to the quantity and disposition of the osseous matter going to form it, and which is deposited sometimes in layers and sometimes in grains. The quantity of the contained liquid varied, in the cases observed by Dr. V., from one scruple to seven ounces and a half; it was generally dark and fluid, and never odorous. When the cephalæmatome terminates by resolution, its osseous ring gradually increases from the circumference towards the centre of the tumour, the fluctuation diminishes, and the fluid is absorbed, little by little, until there is left upon the surface of the head only a slightly rounded boss or rising. Such, at least, was the course pursued in one case noted by Dr. V., and the partial details furnished by other authors seem to establish it as a general rule. When it is considered expedient to employ active treatment for the removal of the tumour, the lancet gives the surest and most prompt results. An incision should be made for evacuating the contents, taking care to avoid wounding the arteries which ramify over the surface of the tumour. The operation is unattended with any risk, for the extreme vitality of the bone renders gangrene nearly impossible, and favours the speedy adhesion to it of the integuments.

*Œdema of new-born Infants.*—Under this name, Dr. Valleix describes one of the most common causes of death, at the Foundling Hospital of Paris. It generally attacks infants of a feeble constitution, or those born before term, and that within three or four days after birth. The invasion of the disease is indicated by a general violet tint of the skin, a coldness of the whole body, and particularly of the extremities; a strong inclination to sleep, and, finally, by short, quick, inspirations, separated by long intervals. To these phenomena succeeds an œdema of the hands and feet, greatest on the side upon which the patient reclines, and gradually becoming general. The skin now becomes livid, and as death approaches, the œdema invades the chest, the coldness of the skin grows intense, and a bloody froth stands upon the lips. The lesions found after death consist in an infiltration of the subcutaneous cellular tissue by yellow serum. The intermuscular tissue retains its natural qualities. The heart, and the entire circulatory system, arteries as well as veins, is found gorged with dark and fluid blood; the distension of the veins, particularly, is sometimes enormous. The only treatment mentioned, possessing any distinct advantage, is the application of leeches to the anus, or behind the ears.

